# Associations of muscle size and fatty infiltration with bone mineral density of the proximal femur bone

**DOI:** 10.3389/fendo.2022.990487

**Published:** 2022-09-27

**Authors:** Junfei Li, Yijing Wang, Xuesong Zhang, Ping Zhang, Yunshan Su, Lin Bai, Yali Wang, Ming Wang, Jian Zhao

**Affiliations:** ^1^ Department of Radiology, The Third Hospital of Hebei Medical University, Shijiazhuang, China; ^2^ Department of Radiology, Hebei General Hospital, Shijiazhuang, China; ^3^ Department of Pediatric Orthopedics, The Third Hospital of Hebei Medical University, Shijiazhuang, China

**Keywords:** muscle cross-sectional area, water/fat imaging, quantitative computed tomography (QCT), proximal femur, bone mineral density (BMD)

## Abstract

**Purpose:**

To investigate the relationship of muscle atrophy and fat infiltration around the hip joint with areal bone mineral density (aBMD) in each subregion of the proximal femur.

**Materials and methods:**

In total, 144 participants (66 women and 78 men) were examined by quantitative computed tomography (QCT), and areal bone mineral density (aBMD) of the femoral neck (FN), trochanter (TR), and intertrochanter (IT) of the proximal femur were obtained. The cross-sectional area (CSA) and proton density fat fraction (PDFF) of the gluteus maximus (G.MaxM), gluteus medius (G.MedM), gluteus minimus (G.MinM), and iliopsoas (IliopM) were obtained *via* magnetic resonance imaging (MRI) using the mDIXON-Quant sequence. A multivariate generalized linear model was used to evaluate the correlation of the CSA and PDFF of muscles with aBMD in all subregions of the proximal femur.

**Results:**

The FN integral (Int) aBMD was significantly associated with the G.MaxM CSA (men: *P* = 0.002; women: *P* = 0.008) and PDFF (men: *P <* 0.001; women: *P* = 0.047). Some muscle indexes were related to the FN aBMD in males or females, including the CSA of G.MedM, G.MinM, and IliopM as well as the PDFF of IliopM and G.MinM. Associations of hip muscle parameters with the TR Int aBMD in both males and females were observed, including G.MaxM CSA (men: *P* < 0.001; women: *P* = 0.028) and G.MaxM PDFF (men: *P* = 0.031; women: *P* = 0.038). Other muscle indexes, including G.MedM and IliopM, were related to the TR aBMD, mainly affecting the aBMD of TR cortical (Cort) and TR Int. The IT Int aBMD and IT Cort aBMD showed significant correlation with the muscle indexes of G. MaxM, IliopM, and G.MedM, including the PDFF and CSA in males and females. Further, more indicators of the G.MedM and IliopM correlated with the TR and IT aBMD compared to the FN aBMD.

**Conclusions:**

The CSA of gluteus muscles and iliopsoas had a positive association with the aBMD in the proximal femur, and the PDFF of gluteus muscles and iliopsoas had a negative correlation with the aBMD in the proximal femur. In addition, there was an interaction of the proximal femur aBMD with the muscle size and fatty infiltration of hip muscles.

## Introduction

Osteoporosis is characterized by the absence of trabeculae and cortical bone, which can be diagnosed on the basis of low bone mineral density (BMD). Bone and muscle are closely related in embryogenesis, growth, and aging, and the interaction between bone and muscle is not only based on the mechanical loads and physical forces generated by muscle contraction but also on endocrine factors (1, 2). With the increase of age, osteoporosis is often accompanied by sarcopenia, which has been shown to be associated with low BMD (3, 4). Many studies have demonstrated that changes in bone mass are closely associated with changes in muscle mass. A positive correlation exists between bone and muscle, with a higher lean body mass associated with increased BMD and vice versa (5–7). Fatty infiltration of muscle and bone is known to contribute to sarcopenia and osteoporosis, most likely related to the negative impact of the secretion of inflammatory cytokines by both bone marrow and body fat in a process known as lipotoxicity (8, 9). Osteoporosis is the most important risk factor for fragility fractures, and osteoporotic fragility fracture of the hip is one of the most common fracture types. Reduced muscle mass and function leads to falls and a higher rate of hip fractures. Osteoporosis and reduced skeletal muscle mass are important risk factors for brittle hip fractures in the elderly (10, 11). Most previous studies have independently assessed muscle and fatty infiltration based on dual energy X-ray absorptiometer (DXA), while the direct relationship of muscle size and intramuscular adipose tissue with the proximal femur BMD has not been elucidated.

The modern Dixon technique uses water/fat separation magnetic resonance imaging (MRI) based on chemical shift, which quantifies intramuscular adipose tissue and shows good consistency with magnetic resonance spectroscopy (MRS) (12). However, MRI not only visualizes the anatomical structure but also quantifies the proton density fat fraction (PDFF) with good spatial resolution, short acquisition time, and accurate fat quantification (13, 14). Computed tomography X-ray absorptiometry (CTXA) is a QCTPro (Mindways Inc., Austin, TX) scanning analysis module, which generates two-dimensional (2D) images from three-dimensional (3D) images of the proximal femur region of interest (ROI) and evaluates the areal BMD (aBMD) of integral (Int), trabecular (Trab), and cortical (Cort) bone by region (femoral neck, FN; trochanter, TR; intertrochanter, IT) (15). In contrast to DXA, quantitative computed tomography (QCT) distinguishes cortical bone from trabecular bone. For the measurement of aBMD in the proximal femur, QCT aBMD has good consistency with DXA (16, 17).

To investigate the relationship between muscle atrophy and fatty infiltration around the hip joint and aBMD in the proximal femur, we used a six-echo chemical shift encoded water-fat MRI (mDIXON-Quant, Philips Healthcare) to assess the muscle PDFF representing the proportion of adipose tissue in muscle and the cross-sectional area (CSA) representing muscle volume around the hip joint. QCT was used to calculate the aBMD of the proximal femur. In this prospective cross-sectional study, we examined the correlation of PDFF and CSA of the gluteus maximus (G.MaxM), gluteus medius (G.MedM), gluteus minimus (G.MinM), and iliopsoas muscle (IliopM) with the QCT assessment of the aBMD of FN, TR, and IT, respectively, in middle-aged and elderly subjects.

## Materials and methods

### Study participants

**Figure 1:**
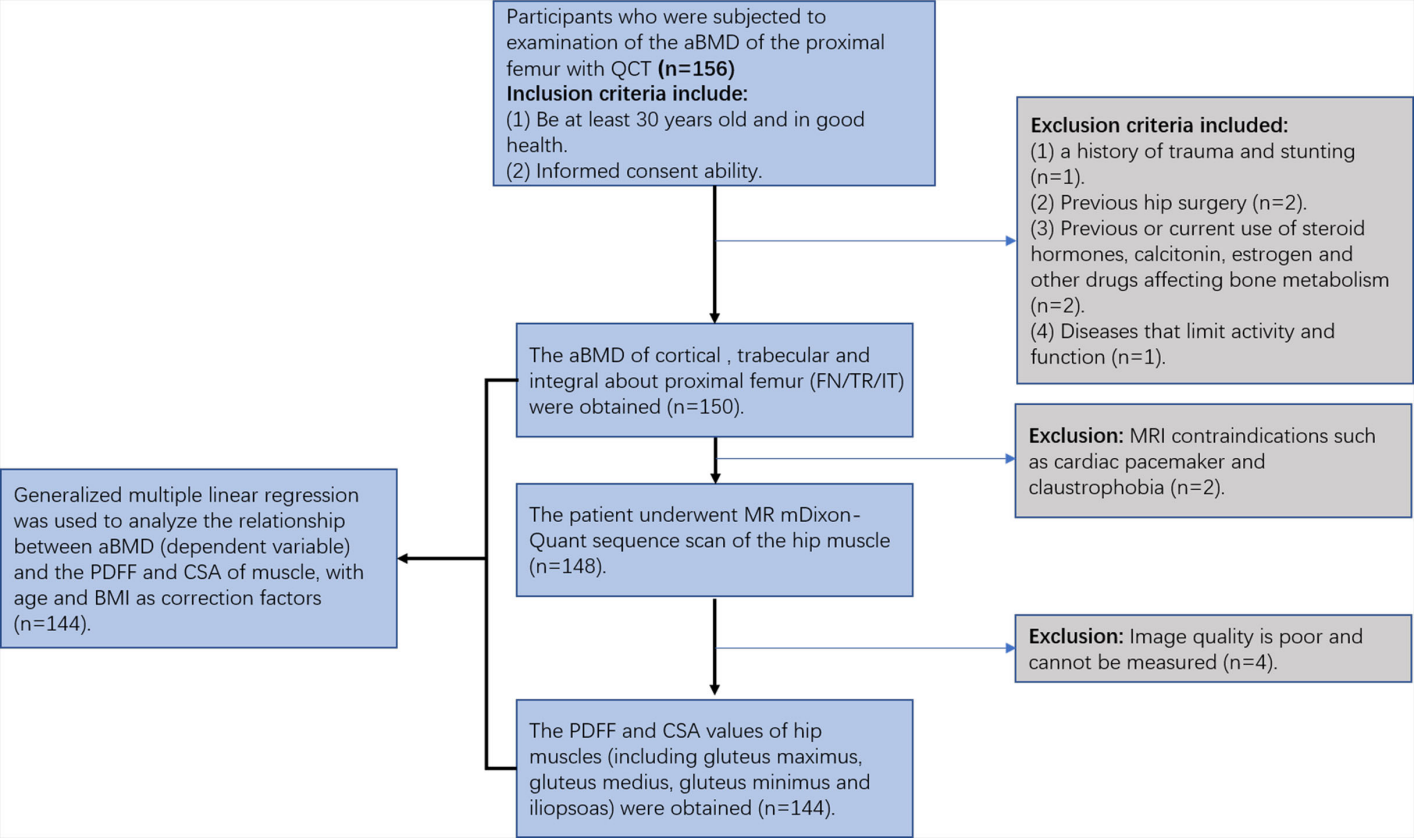
Flow chart of the patient population included in the present study.

Participants who were subjected to examination of the proximal femur aBMD were recruited from the physical examination center of our hospital. The study was approved by the Ethics Committee of The Third Hospital of Hebei Medical University. Informed consent was obtained for each participant. The inclusion criteria were as follows: 1) at least 30 years old and in good health; and 2) no MRI contraindications, such as cardiac pacemaker and claustrophobia. The exclusion criteria were as follows: 1) hip tumor; 2) a history of trauma and stunting; 3) previous hip surgery; 4) previous or current use of steroid hormones, calcitonin, estrogen, and other drugs affecting bone metabolism; and 5) diseases that limit activity and function (**Figure 1**).

### MRI examination

All hip MRI examinations were performed on the same 3.0T MR scanner (Ingenia CX, Phillips, Amsterdam, Netherlands) using a 32-channel torso coil. The MRI sequence parameters of water/fat based on chemical shift coding (mDIXON Quant, Philips Healthcare) were as follows: axial = fast field echo (FFE); repetition time (TR) = 8 ms; echo time (TE) 1 = 1.15 ms; echo spacing = shortest; field of view = 400 × 267 × 325 mm; matrix size = 376 × 299; voxel = 2.5 × 1.5 × 3.0 mm; slice thickness = 5 mm; flip angle = 3°; number of signal averaged (NSA) = 1; and acquisition time = 48 s. Six echoes were used for the quantification of PDFF. All MR images were reviewed and analyzed by a radiologist (XSZ).

**Figure 2:**
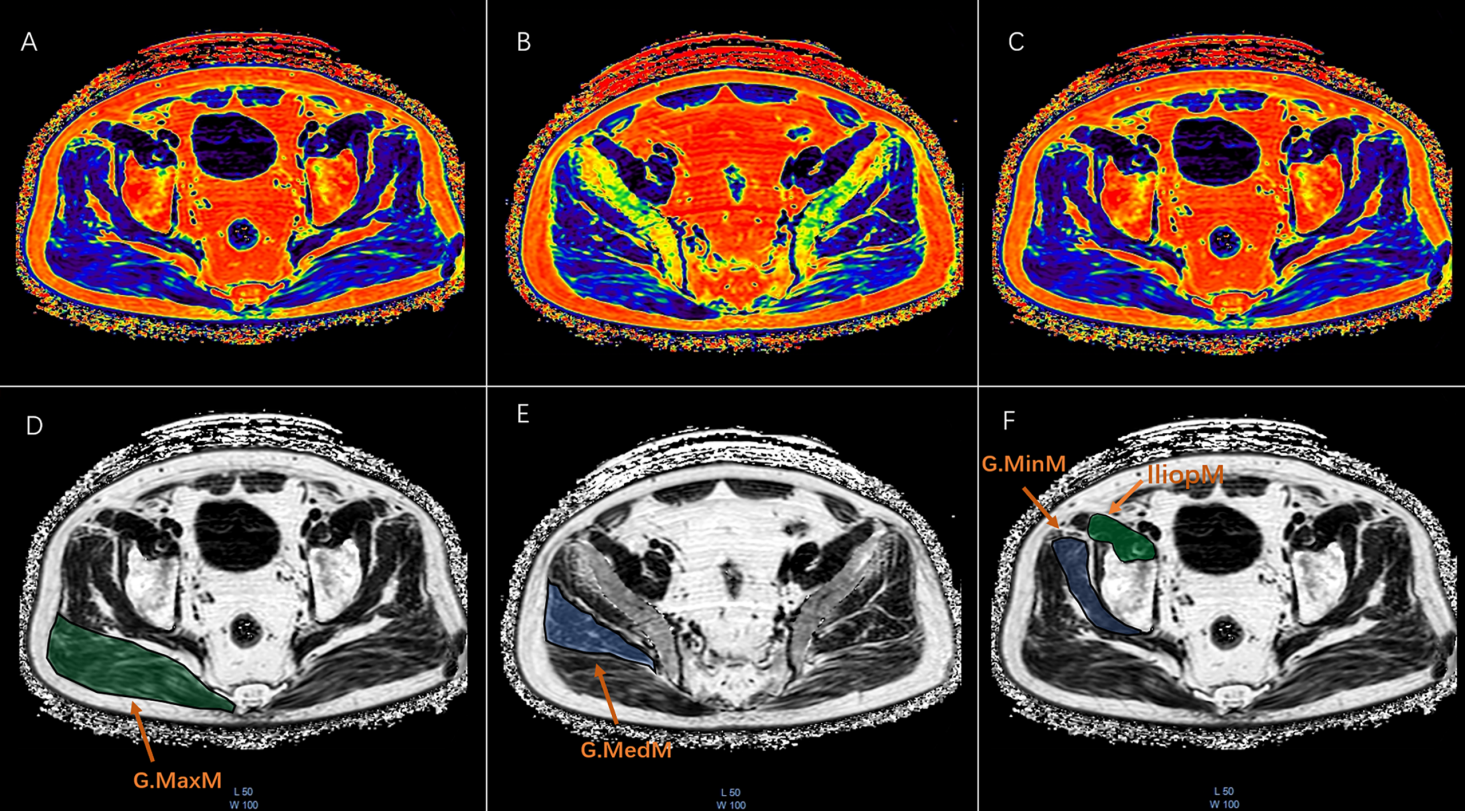
The ROI drawn freehand was placed at the maximum CSA of the fat fraction plot on the axial map of the right gluteus maximus (G.MaxM), gluteus medius (G.MedM), gluteus minimus (G.MinM), and iliopsoas muscle (IliopM). Each ROI was plotted along the edge of the muscle to obtain the PDFF and CSA as shown in the pseudocolor and FF maps of G.MaxM **(A ,D)**, G.MedM **(B, E)**, G.MinM, and IliopM **(C, F)**.

All muscle measurements were performed by the same investigator (XSZ) with more than 2 years of experience who was unaware of the aBMD results. The MR images of water/fat based on chemical shift coding were transferred to the post-processing workstation (InterlliSpace™ Portal, ISP, Philips), and PDFF maps were automatically generated. Seven fat peaks were modeled and T2* corrected. The CSA and PDFF of muscles were measured at the maximum CSA level on the axial fat fraction maps. The G.MaxM at the level of the lower margin of the fourth sacral vertebra and the G.MedM at the level of the first sacral vertebra were analyzed. The G.MinM and IliopM at the level of 0.5 - 1.5 cm at the upper acetabulum margin were analyzed. Free-hand drawn ROIs were separately placed in the axial view of the right sides of the G.MaxM, G.MedM, G.MinM, and IliopM on the fat fraction maps. Each ROI was drawn along the margin of the muscle and outlined muscle contours (**Figure 2**). The PDFF and CSA of G.MaxM, G.MedM, G.MinM, and IliopM were obtained directly. After completing ROI delineation, the PDFF and CSA of G.MaxM, G.MedM, G.MinM, and IliopM were directly obtained from the fat fraction map. Approximately 3 min were required to draw all the ROIs of hip muscles for each subject.

In addition, 20 subjects’ images were randomly selected from the entire data set to evaluate the intra- and inter-reader reliability by a second radiologist (JFL) with more than 2 years of experience. For evaluating the consistency and reliability of different observers, intraclass correlation coefficient (ICC) was determined as follows: ICC < 0.4, poor consistency; 0.4 < ICC < 0.75, moderate consistency; and ICC > 0.75, good consistency. For the bilateral test, a test level of α = 0.05 was used. Good intra-observer (intra-class correlation coefficients, ICC 0.889–0.978, P < 0.001) and moderate inter-observer (ICC 0.693–0.971, P < 0.001) agreements of the muscle measures were found.

### QCT examination

**Figure 3:**
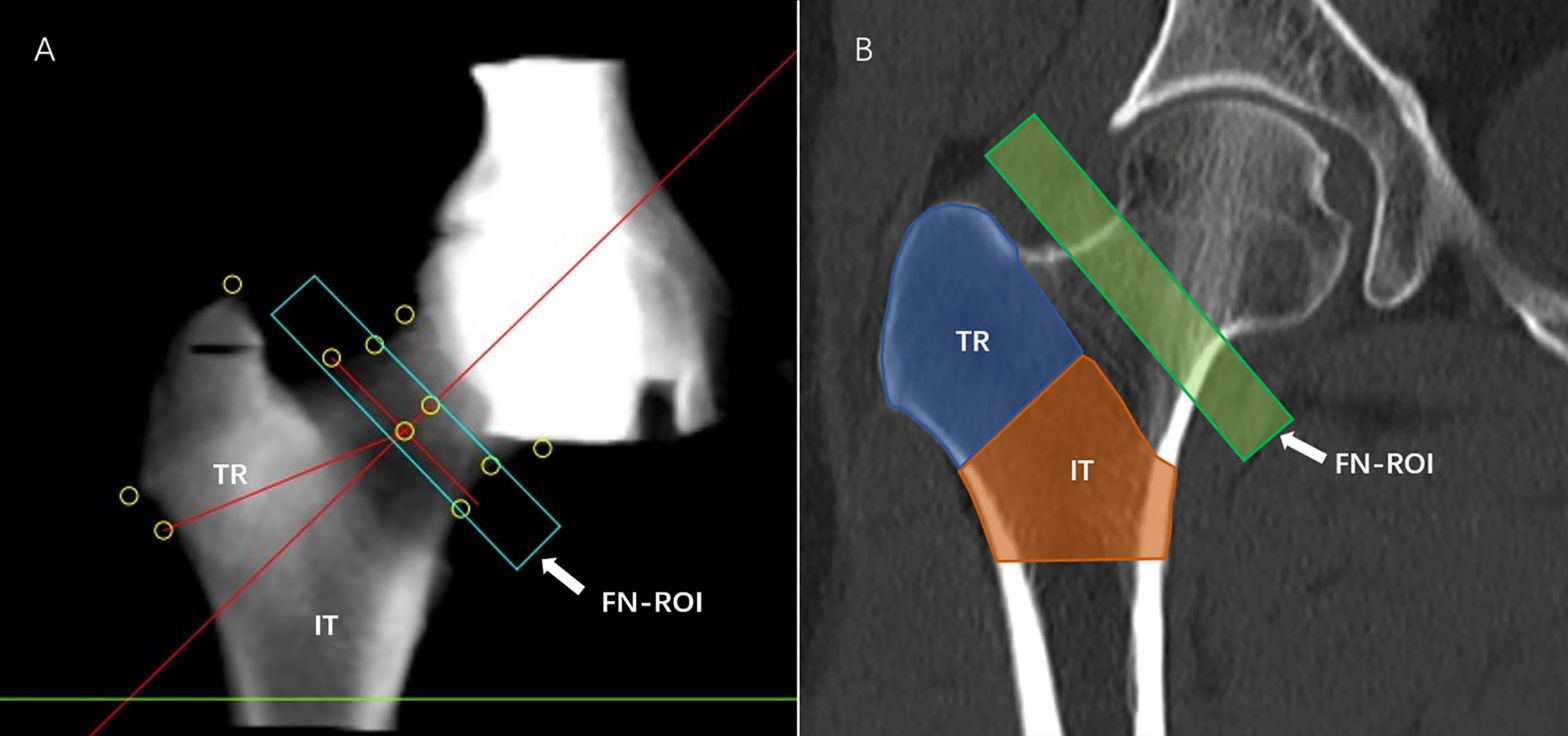
aBMD measurement. QCTPro CTXA was used to analyze the aBMD of the proximal femur, including the FN, TR, and IT **(A, B)**.

CT scans of subjects’ hip joints were performed on a 64-row Siemens Somatom Definition CT scanner (Siemens, Erlangen, Germany) with a Mindways calibrated body model (Mindways Software Inc., Austin, Texas, USA). The acquisition parameters were as follows: 120 kV; 217 mAs; pitch of 1.2; reconstruction slice thickness of 1 mm; reconstruction field of view 50 cm; and medium reconstruction kernel (B40f). The scanning range was 1 cm above the femoral head to 3 cm below the lesser trochanter. The subjects’ knees were flat, and their feet were rotated inwards to reduce overlap between the proximal femur and the acetabulum on the 2D projected image. This study analyzed hip CT scans using the commercial QCTPro (Mindways Inc., Austin, Texas, USA) CTXA module. Three standard DXA hip ROIs were generated, namely, FN, TR, and IT, and DXA equivalent aBMD results of each ROI were obtained. The FN ROI was a narrow frame 10 or 15 mm wide to avoid the overlap between the acetabulum and FN in 2D projection images (**Figure 3**). In the present study, the World Health Organization (WHO) BMD criteria for osteoporosis were used as follows: osteoporosis was defined by a BMD T-score of -2.5 or less at the FN or total hip; osteopenia was defined by a BMD T-score between -1.0 and -2.5 at the FN or total hip; and normal was defined by a BMD T-score of -1 or more at the FN or total hip.

### Statistical analyses

Statistical analyses were performed using SPSS Statistics (IBM, version 26) with a significance level of 0.05. For data with normal distribution, the two independent samples *t*-test was used for comparison between men and women. For data with non-normal distribution, the two-sample Mann-Whitney U test was used to analyze the differences between men and women. The analysis was stratified by sex due to differences in underlying pathological mechanisms of changes in aBMD, muscle CSA, and PDFF between men and women. Generalized multiple linear regression was used to analyze the relationship of the aBMD (dependent variable) of the FN, TR, and IT with the PDFF and CSA of the G.MaxM, G.MedM, G.MinM, and IliopM. To improve various muscle indexes were measured and transferred using sex-specific SD, respectively. Age and BMI were used as correction factors.

## Results

### Study sample characteristics

In total, 144 healthy subjects were included for analysis as shown in **Table 1**. There were 78 males, including 33 subjects with normal BMD, 33 subjects with osteopenia, and 12 subjects with osteoporosis, and there were 66 females, including 20 subjects with normal BMD, 22 subjects with osteopenia, and 24 subjects with osteoporosis. Men had higher a CSA in the G.MaxM (4372.21 vs. 3463.33 mm^2^), G.MedM (2997.23 vs. 2269.58 mm^2^), G.MinM (1149.31 vs. 811.15 mm^2^), and IliopM (1697.37 vs. 1020.38 mm^2^) than women (*P* < 0.05). The PDFF of the G.MaxM (17.86 vs. 21.53%) and IliopM (8.69 vs. 11.15%) in men was lower than that in women (*P* < 0.05). Women also had a higher PDFF in the G.MedM (14.65 vs. 13.92%) and G.MinM (11.67 vs. 10.41%) in men, but there was no statistical difference (*P* > 0.05). Women had a lower aBMD at some sites, including the FN Trab (0.20 vs. 0.23 g/cm^2^), FN Int (0.62 vs. 0.67 g/cm^2^), TR Cort (0.32 vs. 0.36 g/cm^2^), TR Trab (0.16 vs. 0.31 g/cm^2^), TR Int (0.47 vs. 0.67 g/cm^2^), IT Cort (0.52 vs. 0.78 g/cm^2^), IT Trab (0.25 vs. 0.29 g/cm^2^), and IT Int (0.78 vs. 1.05 g/cm^2^) compared to men (*P* < 0.05), but there was no statistical difference for the FN Cort (0.42 vs. 0.45 g/cm^2^, *P* > 0.05).

**Table 1:** Clinical characteristics of the subjects^b^.

Characteristics (Mean ± SD)		Males (*N* = 78)	Females (*N* = 66)	*P*-Value
Age (years)		57.81 ± 12.07	61.88 ± 10.93	**0.032**
Height (cm)		171.74 ± 0.50	160.18 ± 0.69	**<0.001**
Weight (kg)		76.56 ± 11.24	65.41 ± 10.24	**<0.001**
BMI (kg/m^2^)		25.93 ± 0.40	25.44 ± 0.41	0.388
**CSA(mm^2^)**				
G.MaxM		4372.21 ± 1066.84	3463.33 ± 754.64	**<0.001**
G.MedM		2997.23 ± 701.84	2269.58 ± 591.70	**<0.001**
G.MinM		1149.31 ± 264.22	811.15 ± 195.66	**<0.001**
IliopM		1697.37 ± 257.08	1020.38 ± 328.68	**<0.001**
**PDFF (%)**				
G.MaxM		17.86 ± 6.17	21.53 ± 6.49	**0.001**
G.MedM		13.92 ± 4.77	14.65 ± 4.75	0.412
G.MinM		10.41 ± 5.33	11.67 ± 6.13	0.343
IliopM		8.69 ± 3.33	11.15 ± 3.83	**<0.001**
**FN aBMD**(g/cm^2^)	Cort	0.445 ± 0.434	0.423 ± 0.126	0.184
	Trab	0.228 ± 0.057	0.197 ± 0.525	**0.001**
	Int	0.667 ± 0.105	0.616 ± 0.148	**0.009**
**TR aBMD**(g/cm^2^)	Cort	0.361 ± 0.072	0.321 ± 0.093	**0.023**
	Trab	0.313 ± 0.059	0.157 ± 0.063	**<0.001**
	Int	0.673 ± 0.111	0.467 ± 0.117	**<0.001**
**IT aBMD**(g/cm^2^)	Cort	0.776 ± 0.131	0.524 ± 0.129	**<0.001**
	Trab	0.285 ± 0.048	0.247 ± 0.058	**<0.001**
	Int	1.054 ± 0.154	0.774 ± 0.153	**<0.001**

The independent-samples **t** test was used. Most of the remaining data were non-normally distributed, and Mann-Whitney U test was used. (SD, standard deviance; BMI, body mass index; PDFF, proton density fat fraction; CSA, cross-sectional area; G.MaxM, gluteus maximus muscle; G.MedM, gluteus medius muscle; G.MinM, gluteus minimus muscle; IliopM, iliopsoas muscle; aBMD, areal bone mineral density; FN, femoral neck; TR, trochanter; IT, intertrochanter; Int, integral; Trab, trabecular; Cort, cortical). ^b^The bold type indicates statistical difference (P < 0.05).

### FN aBMD

The adjusted beta coefficients and 95% confidence interval (CI) of the FN aBMD sites (FN Int, FN Trab, and FN Cort aBMD) with continuous muscle indexes per sex-specific standard deviation (SD) increase are shown in **Table 2** and **Table 3**. The FN Int aBMD was significantly associated with the G.MaxM CSA (men: *P* = 0.002; women: *P* = 0.008) and PDFF (men: *P* < 0.001; women: *P* = 0.047). There were associations between the FN Cort aBMD and the CSA of G.MaxM (men: *P* = 0.002; women: *P* = 0.002) and G.MaxM PDFF (men: *P* < 0.049; women: *P* = 0.003). In men, 0.194 and -0.196 g/cm^2^ of FN Int aBMD increased with one SD increase of G.MedM area (95% CI, 0.018, 0.370; *P* = 0.031) and PDFF of IliopM (95% CI, -0.348, -0.045; *P* = 0.011), but this significance was not observed in women. The FN Cort aBMD negatively correlated with the IliopM PDFF in men (*β*, -0.240; 95%CI, -0.442, 0.037; *P* = 0.020) and G.MinM PDFF in women (*β*, -0.329; 95%CI, -0.599, -0.058; *P* = 0.017). Associations between the FN Trab aBMD and the CSA of G.MinM (*P* = 0.018) and IliopM (*P* = 0.008) were found only in women.

**Table 2:** Generalized multiple linear standardized regression coefficients and 95% CIs between the aBMD of the FN and various muscle indexes^a,b,c^ in males.

Males						
Variables	FN Int aBMD (g/cm^2^)		FN Cort aBMD (g/cm^2^)		FN Trab aBMD (g/cm^2^)	
CSA (mm^2^)	β (95% CI)	P	β (95% CI)*10^-3^	P	β (95% CI)*10^-3^	P
G.MaxM	0.308(0.109, 0.507)	**0.002**	0.413(0.147, 0.679)	**0.002**	0.246(-0.057, 0.549)	0.112
G.MedM	0.194(0.018, 0.370)	**0.031**	0.086(-0.149, 0.321)	0.472	0.091(-0.176, 0.358)	0.505
G.MinM	0.061(-0.105, 0.227)	0.470	-0.149(-0.370, 0.072)	0.187	0.198(-0.054, 0.450)	0.124
IliopM	-0.080(-0.225, 0.065)	0.279	-0.078(-0.272, 0.115)	0.427	0.037(-0.184, 0.257)	0.744
**PDFF(%)**						
G.MaxM	-0.362(-0.541, -0.182)	**<0.001**	-0.238(-0.477, 0.001)	**0.049**	-0.103(-0.375, 0.170)	0.460
G.MedM	-0.176(-0.383, 0.030)	0.094	-0.170(-0.446, 0.106)	0.226	-0.013(-0.327, 0.301)	0.936
G.MinM	0.065(-0.086, 0.215)	0.398	0.143(-0.058, 0.343)	0.164	0.107(-0.122, 0.336)	0.361
IliopM	-0.196(-0.348, -0.045)	**0.011**	-0.240(-0.442, -0.037)	**0.020**	-0.203(-0.433, 0.028)	0.085

PDFF, proton density fat fraction; CSA, cross-sectional area; G.MaxM, gluteus maximus muscle; G.MedM, gluteus medius muscle; G.MinM, gluteus minimus muscle; IliopM, iliopsoas muscle; aBMD, areal bone mineral density; FN, femoral neck; Int, integral; Trab, trabecular; Cort, cortical. ^a^Adjusted for age and body mass index (BMI). ^b^ The bold type indicates statistical difference (P < 0.05). ^c^β for standard deviance increase of continuous muscle variables.

**Table 3:** Generalized multiple linear standardized regression coefficients and 95% CIs between the aBMD of the FN and various muscle indexes^a,b,c^ in females.

Females						
Variables	FN Int aBMD (g/cm^2^)		FN Cort aBMD (g/cm^2^)		FN Trab aBMD (g/cm^2^)	
CSA (mm^2^)	β (95% CI)	P	β (95% CI)	P	β (95% CI)	P
G.MaxM	0.435(0.116, 0.754)	**0.008**	0.551(0.196, 0.905)	**0.002**	0.079(-0.279, 0.437)	0.664
G.MedM	-0.033(-0.321, 0.255)	0.823	-0.217(-0.538, 0.103)	0.183	0.175(-0.148, 0.498)	0.287
G.MinM	0.164(-0.057, 0.385)	0.147	0.015(-0.231, 0.260)	0.908	0.299(0.051, 0.547)	**0.018**
IliopM	0.049(-0.201, 0.299)	0.702	-0.079(-0.357, 0.199)	0.578	0.378(0.098, 0.658)	**0.008**
**PDFF(%)**						
G.MaxM	-0.290(-0.577, -0.004)	**0.047**	-0.477(-0.795, -0.159)	**0.003**	0.002(-0.319, 0.322)	0.992
G.MedM	0.075(-0.294, 0.444)	0.689	0.261(-0.149, 0.671)	0.211	0.129(-0.284, 0.542)	0.541
G.MinM	-0.232(-0.476, 0.011)	0.061	-0.329(-0.599, -0.058)	**0.017**	-0.211(-0.484, 0.062)	0.130
IliopM	-0.024(-0.253, 0.205)	0.836	-0.044(-0.299, 0.211)	0.735	0.235(-0.022, 0.492)	0.073

PDFF, proton density fat fraction; CSA, cross-sectional area; G.MaxM, gluteus maximus muscle; G.MedM, gluteus medius muscle; G.MinM, gluteus minimus muscle; IliopM, iliopsoas muscle; aBMD, areal bone mineral density; FN, femoral neck; Int, integral; Trab, trabecular; Cort, cortical. ^a^Adjusted for age and body mass index (BMI). ^b^The bold type indicates statistical difference (P < 0.05). ^c^β for standard deviance increase of continuous muscle variables.

### TR aBMD

The results of generalized linear models for the associations between the TR aBMD and muscle indexes are presented in **Table 4** and **Table 5**. Some muscle indexes were related to the TR Int aBMD in both men and women, including the G.MaxM CSA (men: *P* < 0.001; women: *P* = 0.028) and G.MaxM PDFF (men: *P* = 0.031; women: *P* = 0.038). In addition to the above correlation with the TR Int aBMD, associations with the G.MaxM CSA were found for the TR Cort aBMD (*P* = 0.002) and TR Trab aBMD (*P* = 0.001) in men but not in women. Moreover, the G.MedM CSA was also correlated with the TR Int aBMD (*P* < 0.001), TR Cort aBMD (*P* = 0.047), and TR Trab aBMD (*P* = 0.017) in men. In women, 0.404 and -0.199 g/cm^2^ of the TR Int aBMD increased with one SD increase of IliopM CSA (95% CI, 0.204, 0.603; *P* < 0.001) and IliopM PDFF (95% CI, -0.382, -0.016; *P* = 0.033), but this significance was not found in men. Other muscle indexes were related to the TR aBMD in women, including the IliopM CSA (*P* = 0.004) to TR Cort aBMD, the G.MaxM PDFF (*P* = 0.014) to TR Cort aBMD, and the IliopM PDFF (*P* = 0.001) to TR Trab aBMD.

**Table 4:** Generalized multiple linear standardized regression coefficients and 95% CIs between the aBMD of the TR and various muscle indexes^a,b,c^ in males.

Males						
Variables	TR Int aBMD (g/cm^2^)		TR Cort aBMD (g/cm^2^)		TR Trab aBMD (g/cm^2^)	
CSA (mm^2^)	β (95% CI)	P	β (95% CI)	P	β (95% CI)	P
G.MaxM	0.473(0.275, 0.672)	**<0.001**	0.422(0.149, 0.695)	**0.002**	0.441(0.183, 0.700)	**0.001**
G.MedM	0.047(-0.128, 0.222)	0.600	0.090(-0.150, 0.331)	0.462	-0.028(-0.256, 0.200)	0.812
G.MinM	-0.168(-0.333, -0.003)	**0.046**	-0.147(-0.373, 0.080)	0.205	-0.017(-0.232, 0.198)	0.876
IliopM	-0.028(-0.173, 0.116)	0.702	0.032(-0.167, 0.230)	0.756	-0.027(-0.215, 0.161)	0.780
**PDFF(%)**						
G.MaxM	-0.197(-0.375, 0.018)	**0.031**	-0.139(-0.384, 0.107)	0.268	-0.217(-0.450, 0.015)	0.066
G.MedM	-0.444(-0.650, -0.238)	**<0.001**	-0.274(-0.557, 0.009)	**0.047**	-0.328(-0.596, -0.060)	**0.017**
G.MinM	0.132(-0.017, 0.282)	0.083	0.032(-0.174, 0.238)	0.761	0.176(-0.019, 0.371)	0.078
IliopM	-0.034(-0.185, 0.116)	0.654	-0.055(-0.262, 0.153)	0.606	-0.020(-0.217, 0.176)	0.841

PDFF, proton density fat fraction; CSA, cross-sectional area; G.MaxM, gluteus maximus muscle; G.MedM, gluteus medius muscle; G.MinM, gluteus minimus muscle; IliopM, iliopsoas muscle; aBMD, areal bone mineral density; TR, trochanter; Int, integral; Trab, trabecular; Cort, cortical. ^a^Adjusted for age and body mass index (BMI). ^b^The bold type indicates statistical difference (P < 0.05). ^c^β for standard deviance increase of continuous muscle variables.

**Table 5:** Generalized multiple linear standardized regression coefficients and 95% CIs between the aBMD of the TR and various muscle indexes^a,b,c^ in females.

Females						
Variables	TR Int aBMD (g/cm^2^)		TR Cort aBMD (g/cm^2^)		TR Trab aBMD (g/cm^2^)	
CSA(mm^2^)	β(95% CI)	P	β(95% CI)	P	β(95% CI)	P
G.MaxM	0.286(0.031, 0.541)	**0.028**	0.192(-0.102, 0.487)	0.201	-0.256(-0.672, 0.160)	0.227
G.MedM	0.033(-0.197, 0.263)	0.779	0.036(-0.231, 0.302)	0.794	0.348(-0.027, 0.724)	0.069
G.MinM	-0.075(-0.252, 0.101)	0.403	-0.177(-0.381, 0.027)	0.090	-0.094(-0.382, 0.194)	0.173
IliopM	0.404(0.204, 0.603)	**<0.001**	0.340(0.109, 0.571)	**0.004**	-0.206(-0.119, 0.532)	0.214
**PDFF(%)**						
G.MaxM	-0.241(-0.470, 0.013)	**0.038**	-0.331(-0.595, -0.067)	**0.014**	0.267(-0.106, 0.639)	0.161
G.MedM	0.066(-0.229, 0.360)	0.662	0.310(-0.030, 0.651)	0.074	0.074(-0.406, 0.554)	0.763
G.MinM	-0.050(-0.244, 0.144)	0.615	-0.121(-0.346, 0.104)	0.291	-0.221(-0.538, 0.096)	0.173
IliopM	-0.199(-0.382, -0.016)	**0.033**	-0.139(-0.350, 0.073)	0.199	-0.493(-0.791, -0.194)	**0.001**

PDFF, proton density fat fraction; CSA, cross-sectional area; G.MaxM, gluteus maximus muscle; G.MedM, gluteus medius muscle; G.MinM, gluteus minimus muscle; IliopM, iliopsoas muscle; aBMD, areal bone mineral density; TR, trochanter; Int, integral; Trab, trabecular; Cort, cortical. ^a^Adjusted for age and body mass index (BMI). ^b^The bold type indicates statistical difference (P < 0.05). ^c^β for standard deviance increase of continuous muscle variables.

### IT aBMD

**Table 6** and **Table 7** show the results from the multivariate generalized linear models, assessing the associations of the IT aBMD with eight muscle indexes, including the CSA and PDFF of the G. MaxM, G.MedM, G.MinM, and IliopM. The IT Int aBMD showed a significant association with the G. MaxM CSA (men: *P* = 0.005; women: *P* < 0.001), G. MaxM PDFF (only women, *P* = 0.041), IliopM CSA (only men, *P* = 0.007), IliopM PDFF (men: *P* = 0.001; women: *P* = 0.003), G.MedM CSA (only women, *P* < 0.001), and G.MedM PDFF (only women, *P* = 0.038). Associations with the IT Cort aBMD were found for the G.MaxM CSA in men (*P* = 0.014), G.MedM CSA in women (*P* = 0.022), IliopM CSA in men (*P* = 0.039), G.MedM PDFF in women (*P* = 0.010), and IliopM PDFF in women (*P* = 0.001). No significance was found for the IT Trab aBMD (all *P* > 0.05).

**Table 6:** Generalized multiple linear standardized regression coefficients and 95% CIs between the aBMD of the IT and various muscle indexes^a,b,c^ in males.

Males						
Variables	IT Int aBMD (g/cm^2^)		IT Cort aBMD (g/cm^2^)		IT Trab aBMD (g/cm^2^)	
CSA (mm^2^)	β (95% CI)	P	β (95% CI)	P	β (95% CI)	P
G.MaxM	0.319(0.096, 0.542)	**0.005**	0.317(0.065, 0.568)	**0.014**	0.008(-0.328, 0.344)	0.964
G.MedM	0.142(-0.055, 0.339)	0.158	0.192(-0.030, 0.414)	0.091	-0.041(-0.337, 0.256)	0.787
G.MinM	-0.077(-0.262, 0.109)	0.417	-0.177(-0.386, 0.032)	0.097	0.168(-0.112, 0.447)	0.240
IliopM	0.224(0.061, 0.386)	**0.007**	0.193(0.009 0.376)	**0.039**	0.096(-0.149, 0.340)	0.443
**PDFF(%)**						
G.MaxM	-0.091(-0.291, 0.110)	0.375	-0.036(-0.262, 0.190)	0.755	-0.071(-0.373, 0.231)	0.645
G.MedM	-0.102(-0.333, 0.130)	0.389	-0.062(-0.323, 0.199)	0.643	-0.244(-0.593, 0.104)	0.169
G.MinM	0.107(-0.061, 0.276)	0.212	0.060(-0.130, 0.250)	0.537	0.117(-0.136, 0.371)	0.364
IliopM	-0.296(-0.466, -0.127)	**0.001**	-0.315(-0.506, -0.123)	**0.001**	-0.105(-0.360, 0.151)	0.422

PDFF, proton density fat fraction; CSA, cross-sectional area; G.MaxM, gluteus maximus muscle; G.MedM, gluteus medius muscle; G.MinM, gluteus minimus muscle; IliopM, iliopsoas muscle; aBMD, areal bone mineral density; IT, intertrochanter; Int, integral; Trab, trabecular; Cort, cortical. ^a^Adjusted for age and body mass index (BMI). ^b^The bold type indicates statistical difference (P < 0.05). ^c^β for standard deviance increase of continuous muscle variables.

**Table 7:** Generalized multiple linear standardized regression coefficients and 95% CIs between the aBMD of the IT and various muscle indexes^a,b,c^ in females.

Females						
Variables	IT Int aBMD (g/cm^2^)		IT Cort aBMD (g/cm^2^)		IT Trab aBMD (g/cm^2^)	
CSA (mm^2^)	β (95% CI)	P	β (95% CI)	P	β (95% CI)	P
G.MaxM	0.363(0.160, 0.567)	**<0.001**	0.177(-0.117, 0.471)	0.238	0.069(-0.296, 0.435)	0.711
G.MedM	0.338(0.155, 0.522)	**<0.001**	0.309(0.044, 0.574)	**0.022**	0.135(-0.195, 0.465)	0.424
G.MinM	0.092(-0.049, 0.233)	0.200	0.161(-0.043, 0.364)	0.122	0.176(-0.077, 0.430)	0.173
IliopM	0.086(-0.074, 0.245)	0.292	-0.030(-0.260, 0.200)	0.797	0.175(-0.112, 0.461)	0.232
**PDFF(%)**						
G.MaxM	-0.190(-0.372, -0.008)	**0.041**	-0.016(-0.279, 0.248)	0.907	-0.288(-0.616, 0.040)	0.085
G.MedM	-0.249(-0.484, -0.014)	**0.038**	-0.445(-0.784, -0.105)	**0.010**	-0.060(-0.483, 0.362)	0.780
G.MinM	0.120(-0.035, 0.275)	0.129	0.194(-0.030, 0.418)	0.090	0.008(-0.271, 0.287)	0.955
IliopM	-0.220(-0.366, -0.074)	**0.003**	-0.342(-0.553, -0.131)	**0.001**	0.048(-0.214, 0.311)	0.719

PDFF, proton density fat fraction; CSA, cross-sectional area; G.MaxM, gluteus maximus muscle; G.MedM, gluteus medius muscle; G.MinM, gluteus minimus muscle; IliopM, iliopsoas muscle; aBMD, areal bone mineral density; IT, intertrochanter; Int, integral; Trab, trabecular; Cort, cortical. ^a^Adjusted for age and body mass index (BMI). ^b^The bold type indicates statistical difference (P < 0.05). ^c^β for standard deviance increase of continuous muscle variables.

## Discussion

The innovation of this study lies in the independent analysis of the relationship of the PDFF and CSA of the muscles around the hip joint with proximal femur aBMD. The present study is the first to demonstrate that both the CSA and PDFF in the muscles around the hip joint are associated with proximal femur aBMD, suggesting that muscle size and fat infiltration in this area influence the proximal femur aBMD. We applied MR-Dixon technology to quantify muscle adipose content, which quantifies muscle adipose tissue, including intramuscular and intermuscular adipose tissue, with high resolution.

The cellular origins of fatty accumulation in muscle arise through several different pathways. One direct route is *via* the accumulation of lipid within myofibers themselves, which is known as intramuscular fat. Another pathway is an accumulation of adipocytes within skeletal muscle, which is known as intermuscular fat (18). Conventional T1-weighted MRI only assesses visible adipose tissue in T1-weighted images, but it is unable to assess small lipid concentrations in localized muscular regions (19). Chemical shift-based water/fat separation methods, including Dixon techniques and the iterative decomposition of water and fat with echo asymmetry and least-squares estimation (IDEAL), overcome the limitations of conventional T1-weighted imaging by allowing high spatial resolution for quantification of adipose tissue in localized regions, including intramuscular and intermuscular lipids. In addition, to obtain true proximal femur aBMD, we used QCT, which has been demonstrated to have good consistency with DXA.

Muscle CSA or muscle thickness, as a simple and practical muscle volume estimation method, has been widely used as an indirect indicator of muscle strength (20, 21). As for the selection of muscle level, the CSA around the muscle abdomen decreases to the greatest extent due to disuse, while the CSA around the muscle end does not significantly change (22, 23). Therefore, to better evaluate the changes of CSA in the muscles around the hip joint, we selected the maximum CSA of the muscle (similar to the muscle abdomen) as the evaluation level. The present study showed that the CSA of the G.MaxM was positively correlated with the aBMD of all subregions of the proximal femur and that it mainly affects the Int aBMD and Cort aBMD of the FN, TR, and IT. The present study also found that the CSA of the IliopM and G.MedM was positively associated with the Int aBMD and Cort aBMD of the TR and IT. Moreover, the cortical shell of long bones is crucial for fracture prevention because it is the main compressive and flexural resistant structure of bone (24, 25). Therefore, the present results suggested that the CSA of the G.MaxM, IliopM, and G.MedM is a protective factor against proximal femoral fractures by influencing proximal femoral aBMD, especially cortical aBMD. The increase in soft tissue thickness mainly dependent on the CSA of muscles in the proximal femur reduces the risk of fracture by reducing the force applied to the femur during lateral falls (26–28), supporting our view from another aspect.

The interaction between bone and muscle is mainly realized by mechanical stimulation and secreted bioactive factors. Mechanical tension caused by muscle initiates osteogenic activity, and both osteoblasts and osteocytes respond to mechanical stimulation. Mechanical transduction also induces cascades of biochemical signals, including the production of hormones and growth factors, which affect the coupling process of bone formation and bone resorption (29). As an indicator of muscle strength, muscle CSA partly reflects the mechanical tension between muscle and bone, suggesting that mechanical stimulation between muscle and bone may be one of the mechanisms by which the CSA of muscle affects the aBMD in the proximal femur. The G.MaxM is the main and strongest muscle of the hip joint, providing the greatest power for the movement of the hip joint and also affecting the aBMD of most regions in the proximal femur. It is possible that the CSA of the IliopM and G.MedM mainly affects the BMD of the TR and IT through mechanical stimulation of muscle attachment.

Fatty infiltration of skeletal muscle is an important manifestation of skeletal muscle aging, which reflects the decrease of skeletal muscle function and muscle strength (30, 31). Previous studies have found that BMD loss is correlated with decreased muscle mass, strength, and function, which is mainly manifested as lean muscle loss and fat infiltration (3, 13). The present results showed that the PDFFs of several muscles were negatively associated with the aBMD of subregions of the proximal femur as follows: the G.MaxM was negatively associated with the aBMD of the FN, TR, and IT; the G.MedM was negatively associated with the aBMD of the TR and IT; and the IliopM was negatively associated with the aBMD of the FN, TR, and IT. Lu (32) found that the lipid infiltration of the G.MaxM and mid-thigh muscle is associated with the aBMD of the proximal femur, which agreed with the present study. Intramuscular fat infiltration impairs the ability of the skeletal muscle to produce normal protein, resulting in decreased insulin sensitivity. Impaired normal protein synthesis leads to reduced muscle strength and muscle atrophy (18, 33). Therefore, muscle fat infiltration in the buttocks reduces the mechanical stimulation to the bone at this site, resulting in a decrease in the BMD of the proximal femur, suggesting that fatty infiltration of muscle negatively affects aBMD at the proximal femur. Moreover, increased fat content of the gluteal muscles contributes to reduced lower extremity performance, conferring increased risk of loss of mobility, falls, and skeletal fractures (34), which is consistent with a previous study showing that reducing muscle fat infiltration and improving muscle strength significantly reduces fracture risk (35, 36).

The present study showed that more indicators of the G.MedM and IliopM correlated with the aBMD of the TR and IT than the aBMD of the FN. Decreased BMD in different areas of the proximal femur may lead to different types of osteoporotic fractures, such as FN fractures or intertrochanteric fractures. It has been reported that the BMD of the TR and IT in the IT fracture group is lower than that in the FN fracture group (37, 38). Wang et al. (36) found that intertrochanteric aBMD is a better predictor of hip fracture than the FN and total hip aBMD, indicating that intertrochanteric aBMD has a better correlation with hip fractures. Thus, increased CSA and decreased PDFF in the G.MedM and IliopM may be protective factors for intertrochanteric fractures. Physical activity and regular exercise reduce muscle fat content, increase muscle CSA, and enhance bone strength, which reduces the risk of hip fractures (18, 39).

The present study found that the PDFF in the hip muscle was negatively correlated with the aBMD in the proximal femur and that the CSA was positively correlated with the aBMD. Skeletal muscle fat infiltration and muscle atrophy coexist with age, which may be due to the different forms of dysfunction in skeletal muscle fibers and the distribution of muscle fiber types in different functional muscle tissues (40, 41). The accumulation of intramuscular lipid with aging is not homogenous across different fiber types. Type I fibers are oxidative slow-twitch fibers that contain high intramuscular lipid content and many mitochondria. In contrast, type II fibers are glycolytic fast-twitch fibers that have low intramuscular lipid content and low aerobic capacity. Type I fibers tend to accumulate more intramuscular lipids with age in human subjects than fast-twitch oxidative fibers (42). The number and degree of atrophy of type II muscle fibers in skeletal muscle of patients with osteoporosis are greater than that of type I muscle fibers, and a significant correlation between the degree of type II myofiber atrophy and proximal femoral BMD has been reported in previous studies (43). The downregulation of IGF-1/PI3K/Akt activity that occurs in osteoporosis may lead to muscle atrophy. Moreover, because IGF-1/PI3K/Akt activity controls glucose uptake in skeletal muscle, the downregulation of this activity may affect mainly glycolytic fibers (type II) due to their capacity of utilizing glucose to produce energy (43, 44). With age, muscles rich in type II fiber atrophy more and accumulate less lipids than muscles rich in type I fiber.

There were several limitations to this study. First, because this study was a cross-sectional study, we were unable to explore the longitudinal relationship between muscle and the proximal femur aBMD. Second, the study population consisted of middle-aged and elderly non-hip disease individuals from one center, which may limit the generalization of the results to other ethnic groups and other age groups. Third, due to the small number of participants in this study, the differences between male and female indicators were not further discussed. Studies with larger samples are needed to further compare the differences between men and women as well as to obtain additional evidence for the relationship between the muscles around the hip joint and the BMD of the proximal femur. Finally, the lack of data on physical activity and muscle strength may reduce the interpretation of the findings.

In conclusion, the CSA of the gluteus muscle and iliopsoas muscle has a positive association with the proximal femur aBMD, and the PDFF of the gluteus muscle and iliopsoas muscle has a negative correlation with the proximal femur aBMD. A better understanding of the relationship of the PDFF and CSA of the muscle with the proximal femur BMD will help provide a better understanding of the prevention of osteoporosis and related complications. Therefore, the CSA and PDFF of the gluteus muscle and iliopsoas muscle may be important factors and clinically significant targets for the treatment of osteoporosis.

## Data availability statement

The original contributions presented in the study are included in the article/supplementary material. Further inquiries can be directed to the corresponding author.

## Ethics statement

The studies involving human participants were reviewed and approved by Ethics Committee of the Third Hospital of Hebei Medical University. The patients/participants provided their written informed consent to participate in this study.

## Author contributions

JL and YiW participated in manuscript drafting, study design, and statistical analyses. XZ and JL participated in MRI data acquisition. YS and PZ participated in writing (review and editing). LB and YaW participated in QCT measurements. MW and JZ participated in study design and manuscript revisions. All authors contributed to the article and approved the submitted version.

## Conflict of interest

The authors declare that the research was conducted in the absence of any commercial or financial relationships that could be construed as a potential conflict of interest.

## Publisher’s note

All claims expressed in this article are solely those of the authors and do not necessarily represent those of their affiliated organizations, or those of the publisher, the editors and the reviewers. Any product that may be evaluated in this article, or claim that may be made by its manufacturer, is not guaranteed or endorsed by the publisher.

## References

[1] Lang TF. The bone-muscle relationship in men and women. J Osteoporos (2011) 2011:702735. doi: 10.4061/2011/702735 22007336 PMC3189615

[2] Herrmann M Engelke K Ebert R Müller-Deubert S Rudert M Ziouti F. Interactions between muscle and bone-where physics meets biology. Biomolecules (2020) 10:432. doi: 10.3390/biom10030432 PMC7175139 32164381

[3] He H Liu Y Tian Q Papasian CJ Hu T Deng HW. Relationship of sarcopenia and body composition with osteoporosis. Osteoporos Int (2016) 27:473–82. doi: 10.1007/s00198-015-3241-8 26243357

[4] Verschueren S Gielen E O'Neill TW Pye SR Adams JE Ward KA. Sarcopenia and its relationship with bone mineral density in middle-aged and elderly European men. Osteoporos Int (2013) 24:87–98. doi: 10.1007/s00198-012-2057-z 22776861

[5] Demontiero O Boersma D Suriyaarachchi P Duque G. Clinical outcomes of impaired muscle and bone interactions. Clin Rev Bone Miner Metab (2014) 12:86–92. doi: 10.1007/s12018-014-9164-7

[6] Burr DB. Muscle strength, bone mass, and age-related bone loss. J Bone Miner Res (1997) 12:1547–51. doi: 10.1359/jbmr.1997.12.10.1547 9333114

[7] Sjoblom S Suuronen J Rikkonen T Honkanen R Kroger H Sirola J. Relationship between postmenopausal osteoporosis and the components of clinical sarcopenia. Maturitas (2013) 75:175–80. doi: 10.1016/j.maturitas.2013.03.016 23628279

[8] Maugeri D Russo MS Franzé C Motta V Motta M Destro G. Correlations between c-reactive protein, interleukin-6, tumor necrosis factor-alpha and body mass index during senile osteoporosis. Arch Gerontol Geriatr (1998) 27:159–63. doi: 10.1016/s0167-4943(98)00110-1 18653160

[9] Ng A Duque G. Osteoporosis as a lipotoxic disease. Bonekey Osteovision (2010) 7:108–23. doi: 10.1138/20100435

[10] Burge R Dawson-Hughes B Solomon DH Wong JB King A Tosteson A. Incidence and economic burden of osteoporosis-related fractures in the united states, 2005-2025. J Bone Miner Res (2007) 22:465–75. doi: 10.1359/jbmr.061113 17144789

[11] Becker DJ Kilgore ML Morrisey MA. The societal burden of osteoporosis. Curr Rheumatol Rep (2010) 12:186–91. doi: 10.1007/s11926-010-0097-y 20425518

[12] Kumar D Link TM Jafarzadeh SR LaValley MP Majumdar S Souza RB. Association of quadriceps adiposity with an increase in knee cartilage, meniscus, or bone marrow lesions over three years. Arthritis Care Res (Hoboken) (2021) 73:1134–9. doi: 10.1002/acr.24232 PMC7606313 32339414

[13] Zhao Y Huang M Serrano Sosa M Cattell R Fan W Li M. Fatty infiltration of paraspinal muscles is associated with bone mineral density of the lumbar spine. Arch Osteoporos (2019) 14:99. doi: 10.1007/s11657-019-0639-5 31617017

[14] Yoo HJ Hong SH Kim DH Choi JY Chae HD Jeong BM. Measurement of fat content in vertebral marrow using a modified dixon sequence to differentiate benign from malignant processes. J Magn Reson Imaging (2017) 45:1534–44. doi: 10.1002/jmri.25496 27690264

[15] Liu Y Wang L Su Y Brown K Yang R Zhang Y. CTXA hip: the effect of partial volume correction on volumetric bone mineral density data for cortical and trabecular bone. Arch Osteoporos (2020) 15:50. doi: 10.1007/s11657-020-00721-8 32193671

[16] Cheng X Wang L Wang Q Ma Y Su Y Li K. Validation of quantitative computed tomography-derived areal bone mineral density with dual energy X-ray absorptiometry in an elderly Chinese population. Chin Med J (Engl) (2014) 127:1445–9. 24762586

[17] Khoo BCC Brown K Cann C Zhu K Henzell S Low V. Comparison of QCT-derived and DXA-derived areal bone mineral density and T scores. Osteoporos Int (2009) 20:1539–45. doi: 10.1007/s00198-008-0820-y 19107384

[18] Hamrick MW McGee-Lawrence ME Frechette DM. Fatty infiltration of skeletal muscle: mechanisms and comparisons with bone marrow adiposity. Front Endocrinol (Lausanne) (2016) 7:69. doi: 10.3389/fendo.2016.00069 27379021 PMC4913107

[19] Karampinos DC Baum T Nardo L Alizai H Yu H Carballido-Gamio J. Characterization of the regional distribution of skeletal muscle adipose tissue in type 2 diabetes using chemical shift-based water/fat separation. J Magn Reson Imaging (2012) 35:899–907. doi: 10.1002/jmri.23512 22127958 PMC3292710

[20] Yamauchi K Kato C Kato T. Characteristics of individual thigh muscles including cross-sectional area and adipose tissue content measured by magnetic resonance imaging in knee osteoarthritis: a cross-sectional study. Rheumatol Int (2019) 39:679–87. doi: 10.1007/s00296-019-04247-2 30689015

[21] Cotofana S Hudelmaier M Wirth W Himmer M Ring-Dimitriou W Sänger AM. Correlation between single-slice muscle anatomical cross-sectional area and muscle volume in thigh extensors, flexors and adductors of perimenopausal women. Eur J Appl Physiol (2010) 110:91–7. doi: 10.1007/s00421-010-1477-8 20401666

[22] Yamauchi K Yoshiko A Suzuki S Kato C Akima H Kato T. Muscle atrophy and recovery of individual thigh muscles as measured by magnetic resonance imaging scan during treatment with cast for ankle or foot fracture. J Orthop Surg (Hong Kong) (2017) 25:2309499017739765. doi: 10.1177/2309499017739765 29137564

[23] Yamauchi K Suzuki S Kato C Kato T. Atrophy of individual thigh muscles measured by MRI in older adults with knee osteoarthritis: A cross-sectional study. Ann Phys Rehabil Med (2020) 63:38–45. doi: 10.1016/j.rehab.2019.06.018 31386911

[24] Carpenter RD Sigurdsson S Zhao S Lu Y Eiriksdottir G Sigurdsson G. Effects of age and sex on the strength and cortical thickness of the femoral neck. Bone (2011) 48:741–7. doi: 10.1016/j.bone.2010.12.004 PMC3075958 21168538

[25] Engelke K. Quantitative computed tomography-current status and new developments. J Clin Densitom (2017) 20:309–21. doi: 10.1016/j.jocd.2017.06.017 28712984

[26] Bouxsein ML Szulc P Munoz F Thrall E Sornay-Rendu E Delmas PD. Contribution of trochanteric soft tissues to fall force estimates, the factor of risk, and prediction of hip fracture risk. J Bone Miner Res (2007) 22:825–31. doi: 10.1359/jbmr.070309 17352651

[27] Nielson CM Bouxsein ML Freitas SS Ensrud KE Orwoll ES. Trochanteric soft tissue thickness and hip fracture in older men. J Clin Endocrinol Metab (2009) 94:491–6. doi: 10.1210/jc.2008-1640 PMC2646514 19017753

[28] Dufour AB Roberts B Broe KE Kiel DP Bouxsein ML Hannan MT. The factor-of-risk biomechanical approach predicts hip fracture in men and women: the framingham study. Osteoporos Int (2012) 23:513–20. doi: 10.1007/s00198-011-1569-2 PMC3289518 21344243

[29] Hoyan L Yi-Xian Q. The effects of frequency-dependent dynamic muscle stimulation on inhibition of trabecular bone loss in a disuse model. Bone (2008) 43:1093–100. doi: 10.1016/j.bone.2008.07.253 PMC2642608 18757047

[30] Wang L Yin L Zhao Y Su Y Sun W Chen S. Muscle density, but not size, correlates well with muscle strength and physical performance. J Am Med Dir Assoc (2021) 22:751–9. doi: 10.1016/j.jamda.2020.06.052 32768372

[31] Kumar D Karampinos DC MacLeod TD Lin W Nardo L Li X. Quadriceps intramuscular fat fraction rather than muscle size is associated with knee osteoarthritis. Osteoarthritis Cartilage (2014) 22(2):226–34. doi: 10.1016/j.joca.2013.12.005 PMC3932784 24361743

[32] Yin L Xu Z Wang L Li W Zhao Y Su Y. Associations of muscle size and density with proximal femur bone in a community dwelling older population. Front Endocrinol (Lausanne) (2020) 11:503. doi: 10.3389/fendo.2020.00503 32849289 PMC7399084

[33] Rivas DA McDonald DJ Rice NP Haran PH Dolnikowski GG Fielding RA. Diminished anabolic signaling response to insulin induced by intramuscular lipid accumulation is associated with inflammation in aging but not obesity. Am J Physiol Regul Integr Comp Physiol (2016) 310:R561–9. doi: 10.1152/ajpregu.00198.2015 PMC4867383 26764052

[34] Goodpaster BH Park SW Harris TB Kritchevsky SB Nevitt M Schwartz AV. The loss of skeletal muscle strength, mass, and quality in older adults: the health, aging and body composition study. J Gerontol A Biol Sci Med Sci (2006) 61:1059–64. doi: 10.1093/gerona/61.10.1059 17077199

[35] Wang L Yin L Zhao Y Su Y Sun W Liu Y. Muscle density discriminates hip fracture better than CTXA hip areal bone mineral density. J Cachexia Sarcopenia Muscle (2020) 11:1799–812. doi: 10.1002/jcsm.12616 PMC7749550 32894000

[36] Wang L Yin L Yang M Ge Y Liu Y Su Y. Muscle density is an independent risk factor of second hip fracture: a prospective cohort study. J Cachexia Sarcopenia Muscle (2022) 13:1927–37. doi: 10.1002/jcsm.12996 PMC9178374 35429146

[37] Cho Y Lee I Ha SH Park JH Park JH. Comparison of hip subregion bone mineral density to the type of proximal femur fracture. Arch Osteoporos (2020) 15(1):122. doi: 10.1007/s11657-020-00789-2 32757078

[38] Wu CC Wang CJ Shyu YI. Variations in bone mineral density of proximal femora of elderly people with hip fractures: a case-control analysis. J Trauma (2011) 71:1720–5. doi: 10.1097/TA.0b013e3182185aeb 21841516

[39] Leenders M Verdijk LB van der Hoeven L van Kranenburg J Nilwik R van Loon LJ. Elderly men and women benefit equally from prolonged resistance-type exercise training. J Gerontol A Biol Sci Med Sci (2013) 68:769–79. doi: 10.1093/gerona/gls241 23223011

[40] Hirschfeld HP Kinsella R Duque G. Osteosarcopenia: where bone, muscle, and fat collide. Osteoporos Int (2017) 28:2781–90. doi: 10.1007/s00198-017-4151-8 28733716

[41] Johnson MA Polgar J Weightman D Appleton D. Data on the distribution of fibre types in thirty-six human muscles an autopsy study. J Neurol Sci (1973) 18:111–29. doi: 10.1016/0022-510x(73)90023-3 4120482

[42] Gueugneau M Coudy-Gandilhon C Théron L Meunier B Barboiron C Combaret L. Skeletal muscle lipid content and oxidative activity in relation to muscle fiber type in aging and metabolic syndrome. J Gerontol A Biol Sci Med Sci (2015) 70:566–76. doi: 10.1093/gerona/glu086 24939997

[43] Terracciano C Celi M Lecce D Baldi J Rastelli E Lena E. Differential features of muscle fiber atrophy in osteoporosis and osteoarthritis. Osteoporos Int (2013) 24:1095–100. doi: 10.1007/s00198-012-1990-1 PMC3572370 22535191

[44] Perrini S Laviola L Carreira MC Cignarelli A Natalicchio A Giorgino F. The GH/IGF1 axis and signaling pathways in the muscle and bone: mechanisms underlying age-related skeletal muscle wasting and osteoporosis. J Endocrinol (2010) 205:201–10. doi: 10.1677/JOE-09-0431 20197302

